# Early and Prolonged Mild Hypothermia in Patients with Poor-Grade Subarachnoid Hemorrhage: A Pilot Study

**DOI:** 10.1089/ther.2022.0013

**Published:** 2022-11-25

**Authors:** Jong-Kook Rhim, Jeong Jin Park, Heungcheol Kim, Jin Pyeong Jeon

**Affiliations:** ^1^ Department of Neurosurgery, Jeju National University College of Medicine, Jeju, Republic of Korea.; ^2^ Department of Neurology, Konkuk University Medical Center, Seoul, Republic of Korea.; ^3^ Department of Radiology and Hallym University College of Medicine, Chuncheon, Republic of Korea.; ^4^ Department of Neurosurgery, Hallym University College of Medicine, Chuncheon, Republic of Korea.

**Keywords:** subarachnoid hemorrhage, hypothermia, outcome

## Abstract

We assessed the feasibility of therapeutic early and prolonged mild hypothermia (MH) in patients with poor-grade subarachnoid hemorrhage (SAH). A retrospective pilot study was conducted for poor-grade SAH patients at two university hospitals from March 2015 to December 2018 who had received MH immediately after coil embolization and maintained a target temperature of 34–35°C for 5 days. A matched controlled design at a 1:2 ratio was used to compare MH therapy outcomes. The primary goal was to assess the two groups' severe functional outcomes at discharge defined as a modified Rankin Scale score of 4–6. The secondary aim was to assess mortality and severe vasospasm depending upon MH. A binary logistic regression analysis was performed to identify relevant risk factors for the outcomes. A total of 54 patients (18 with MH treatment and 36 without MH treatment) were included. Severe functional outcome was significantly decreased in poor-grade SAH patients with MH (*n* = 7, 38.9%) than those without MH (*n* = 25, 69.4%; *p* = 0.031). In patients treated with MH, mortality and severe vasospasm tended to be less common, although the difference was not statistically significant. A binary logistic regression analysis revealed that early and prolonged MH (odds ratio [OR] = 0.156, 95% confidence intervals [CI]: 0.037–0.644) and severe vasospasm (OR = 5.593, 95% CI: 1.372–22.812) were risk factors for severe functional outcomes. This study shows potential therapeutic effect of early and prolonged MH treatment in poor-grade SAH patients. A randomized controlled study with a large number of patients is warranted in the future.

## Introduction

Aneurysmal subarachnoid hemorrhage (SAH) remains the most life-threating neurological emergency. SAH accounts for ∼5% of all strokes (Nieuwkamp *et al.*, 2009). By 2010, its global incidence had decreased from 10.2 cases to 6.1 cases per 100,000 person-years since 1980 (Etminan *et al.*, 2019). However, after stroke, various complications may arise with time. Within 72 hours of bleeding, early brain injury (EBI) becomes a major concern. Treatments currently focus on lowering increased intracranial pressure (IICP) and raising cerebral perfusion pressure (CPP).

After that, delayed cerebral ischemia (DCI), mainly caused by cerebral vasospasm, becomes a major concern for clinicians (Foreman, 2016). Anatomically, SAH refers to blood leaking into the cerebrospinal fluid space surrounding the cerebral arteries, causing continuous inflammation around them. Vasospasm in relatively large cerebral arteries can, therefore, occur after a certain amount of time instead of immediately after SAH. EBI and DCI are closely related to each other. One study has reported that inotropic cardiac support improved cerebral blood flow (CBF) caused by EBI, resulting in reduced DCI and better neurological outcomes (Mutoh *et al.*, 2017). Thus, EBI has gradually become a therapeutic target in the medical field to reduce DCI after SAH (Mutoh *et al.*, 2017).

The prognosis for SAH patients is greatly affected by the disease severity at the time of admission. Although poor-grade SAH accounts for ∼20–30% of all SAH cases, its mortality is as high as 45–60%, even if aneurysms are successfully treated (Seule *et al.*, 2009; Zheng *et al.*, 2019). In addition to conventional treatments such as coil embolization and surgical clipping, it is necessary to effectively treat brain damage increased by SAH. Therapeutic hypothermia has become increasingly common in SAH patients in anticipation of neurological recovery and favorable prognoses.

An experimental *in vivo* study has shown that mild hypothermia (MH) can decrease reactive oxygen species and activate apoptosis, thereby protecting against brain damage caused by bleeding (Lv *et al.*, 2016). MH is also known to be safe for use in poor-grade SAH. However, its clinical efficacy remains controversial, although MH is generally effective in cardiopulmonary resuscitation (Arrich *et al.*, 2012). We believe that significant heterogeneity across studies, such as the initiation and duration of MH treatment, might have resulted in conflicting results in patients with poor-grade SAH.

Guidelines on when and how long to administer MH to patients with poor-grade SAH are still unavailable. Theoretically, we hypothesized that initiating MH treatment as soon as possible after aneurysmal rupture and maintaining MH continuously throughout the IICP period as well as the EBI period could reduce adverse outcomes in patients. To confirm our assumptions, we analyzed treatment outcomes of early and prolonged MH in patients with poor-grade SAH focusing on two issues. First, we treated patients with MH within 8 hours of symptom onset. Patients with poor-grade SAH had a better prognosis when the average time from symptom onset to MH was 10 hours (Choi *et al.*, 2017).

Thus, we started MH fewer than 8 hours after symptom onset. Second, we administered MH for 5 days unlike previous studies (Smrcka *et al.*, 2005; Anei *et al.*, 2010; Choi *et al.*, 2017). After symptom onset, neuromonitoring of SAH patients showed decreased mean ICP values at 72 hours before increasing again by 120 hours (Helbok *et al.*, 2015). Thus, we maintained MH for up to 5 days to decrease the CBF caused by IICP. With these two considerations in mind, we performed a pilot study to determine whether early and prolonged MH could improve the prognosis for patients having poor-grade SAH.

## Material and Methods

### Participants

A retrospective pilot study was conducted on SAH patients who visited two university hospitals between March 2015 and December 2018. Inclusion criteria were (1) 18–70 years of age; (2) spontaneous SAH due to aneurysmal rupture; (3) early and prolonged MH administered for 5 days that had started within 8 hours of symptom onset, as well as the time spent repairing the ruptured aneurysm; (4) patients who had undergone coil embolization; and (5) poor-grade SAH on admission as defined by a Hunt and Hess grade of 4 to 5, or a modified Fisher Scale of 3 to 4 (Kim *et al.*, 2018).

Exclusion criteria were (1) patients with disturbed brainstem reflexes such as pupillary light reflex and corneal reflex, (2) nonaneurysmal SAH or angiogram-negative SAH, (3) patients with systematic inflammation response syndrome or sepsis on admission, (4) increased bleeding tendency, (5) patients who had undergone surgical clipping, and (6) patients who had started having SAH-related symptoms at an unknown time (Anei *et al.*, 2010; Kuramatsu *et al.*, 2015; Choi *et al.*, 2017).

We used a matched controlled design to compare outcomes according to early and prolonged MH in patients with poor-grade SAH. Matching procedures were performed at a 1:2 ratio considering different variables such as initial clinical status, hemorrhage amounts, patient age, and underlying diseases (Kuramatsu *et al.*, 2015). We matched a total of 36 patients from a prospective multicenter study database called “The First Korean Stroke Genetics Association Research” (Hong *et al.*, 2022; Youn *et al.*, 2022).

### Study outcomes

Our primary goal was to determine whether early and prolonged MH might decrease severe functional outcomes in poor-grade SAH patients. Our secondary goals were to investigate different effects of MH on mortality or severe vasospasm. We also evaluated adverse events that occurred with prolonged MH. Severe functional outcomes were defined as a modified Rankin Scale (mRS) score of 4–6 at discharge (Choi *et al.*, 2017). We performed transcranial Doppler (TCD) ultrasound daily to screen for blood velocities suggesting cerebral vasospasm. Severe vasospasm was suspected when TCD velocities were >200 cm/s in the middle cerebral artery or 85 cm/s in the basilar artery (Park *et al.*, 2021).

If we suspected severe vasospasm, we conducted imaging tests such as computed tomography angiography (CTA), magnetic resonance angiography, or cerebral angiography to further confirm the severity of the vasospasm. Severe vasospasm was defined as a decrease in the artery diameter ≥70% as compared with the diameter measured from initial imaging tests (Park *et al.*, 2021). In the case of severe vasospasm, nimodipine was administrated through an intra-arterial approach. The procedure was performed in the angio suite under local anesthesia. After confirming vasospasm severity, 1.5–3 mg of nimodipine was administrated through microcatheter placed in the internal carotid artery for 20–30 minutes (Hejcl *et al.*, 2017).

We routinely checked CTA at 3, 7, and 14 days after SAH to identify vasospasm and its severity (Weir *et al.*, 1978; Park *et al.*, 2020, 2021). We reviewed adverse events occurring within 7 days of hospitalization (Choi *et al.*, 2017). We used the previous study's criteria, such as bradycardia, hypotension, pneumonia, and electrolyte disturbances, to define each adverse event (Choi *et al.*, 2017). We reviewed clinical information (e.g., gender, age, underlying medical diseases, and smoking), radiological information, treatments, and adverse events (Jeon *et al.*, 2012). This study was approved by the institutional review boards (IRB Nos.: 2016-31, 2018-6, and 2019-06) of the participating hospitals. Informed consent was obtained from patients or their legal representatives.

### Treatment and hypothermia protocols

After CT imaging confirmed a diagnosis of SAH on admission, all patients underwent cerebrovascular angiography. We performed extraventricular drainage after aneurysm coil embolization. In the intensive care unit, continuous lumbar drainage was used to control IICP. To prevent vasospasm, nimodipine (Samjin Pharmaceutical, Seoul, Korea) was injected intravenously at a dose of 20 μg/[kg·h] (Park *et al.*, 2021). Upon securing the ruptured aneurysm, we induced MH with surface cooling blankets connected to Medi-Therm^®^ III Hyper/Hypothermia Machine MTA6900 (Gaymar industries, Inc., Orchard Park, NY, USA) and ice bags to the groin, axillae, and neck (Seule *et al.*, 2009).

We targeted the temperatures to be between 34°C and 35°C. We did not lower the core body temperature to be <34°C as assessed with a Foley catheter in the urinary bladder. MH patients were sedated using midazolam combined with remifentanil for analgesia (Choi *et al.*, 2017).

Rewarming was carried out slowly with the goal of not increasing the maximal temperature by >0.5°C in a 24-hour period (Kuramatsu *et al.*, 2015; Mader, 2019). Rewarming was usually carried out over 3 or 4 days using a WarmTouch™ (Model 6000; Covidien, Mansfield, MA, USA) or a Bair Hugger warming device (Model 505/10; Augustine Medical, Inc., Eden Prairie, MN, USA) (Wang *et al.*, 2022). Patients having a Bedside Shivering Assessment Scale score >1 were treated with sedation or pethidine infusion and muscle relaxants (Kuramatsu *et al.*, 2015).

### Statistical analysis

Discrete and continuous data are presented as number (percentage) and median (25–75% percentile). Chi-squared or Fisher's exact test and paired sample *T* test were used to compare discrete and continuous variables, respectively. A binary logistic regression model was used to identify relevant risk factors of outcomes. Statistical results are described as an odds ratio (OR) and 95% confidence intervals (CIs). *p*-Value <0.05 was regarded as statistically significant. All statistical analyses were performed using SPSS V.19 (SPSS, Inc., Chicago, IL, USA).

## Results

### Patients enrolled in the study

We enrolled a total of 54 poor-grade SAH patients. Among them, 18 had received early and prolonged MH, whereas 36 of them had not received this. The mean age according to MH did not differ significantly (57.0 ± 8.8 years for patients with MH vs. 56.9 ± 12.7 years for patients without MH, *p* = 0.993). Clinical variables such as hypertension, diabetes mellitus, hyperlipidemia, and smoking did not differ significantly either between the two groups (Table 1). Most cerebral aneurysms were located in the anterior circulation. There was no significant difference in hemoglobin or oxygen saturation in the arterial blood (SaO_2_) between the two groups except for body temperature (Supplementary Fig. S1).

**Table 1.** Differences in Clinical Characteristics, Radiological Findings, Laboratory Results, and Outcomes in Patients with Poor-Grade Subarachnoid Hemorrhage According to Early and Prolonged Mild Hypothermia

Variables	Without MH (*n* = 36)	MH (*n* = 18)	*p*
Clinical characteristics			
Female	21 (58.3%)	12 (66.7%)	0.554
Age, years	56.9 ± 12.7	57.0 ± 8.8	0.993
Hypertension	17 (47.2%)	7 (38.9%)	0.561
Diabetes mellitus	5 (13.9%)	3 (16.7%)	0.786
Hyperlipidemia	6 (16.7%)	4 (22.2%)	0.620
Smoking	4 (11.1%)	3 (16.7%)	0.567
Radiological findings			
Anterior location	31 (86.1%)	16 (88.9%)	0.775
Size (mm)	5.6 ± 1.7	6.5 ± 1.8	0.592
Laboratory results			
Hemoglobin (g/dL)	11.3 ± 1.1	11.4 ± 1.0	0.652
SaO_2_ (%)	94.8 ± 1.8	94.6 ± 1.9	0.791
Outcome			
Severe vasospasm	17 (47.2%)	6 (33.3%)	0.331
Severe functional outcome (mRS score 4–6)	25 (69.4%)	7 (38.9%)	0.031
Mortality	10 (27.8%)	3 (16.7%)	0.368

MH, mild hypothermia; mRS, modified Rankin Scale; SaO_2_, arterial oxygen saturation.

### Risk factors for severe functional outcomes

Poor-grade SAH patients not treated with MH tended to have higher severe vasospasm rates (*n* = 17, 47.2%) and mortality (*n* = 10, 27.8%) than those treated with MH (severe vasospasm, *n* = 6, 33.3%; mortality, *n* = 3, 16.7%). However, their differences were not statistically significant. Severe functional outcomes in poor-grade SAH patients treated with MH were significantly decreased (*n* = 7, 38.9%) compared with those not treated with MH (*n* = 25, 69.4%; *p* = 0.031). The number of patients without MH treatment was as follows: mRS score of 1, *n* = 1 (2.8%); mRS score of 2, *n* = 3 (8.4%); mRS score of 3, *n* = 7 (19.4%); mRS score of 4, *n* = 7 (19.4%); mRS score of 5, *n* = 8 (22.2%); and mRS score of 6, *n* = 10 (27.8%).

**FIG. 1.**
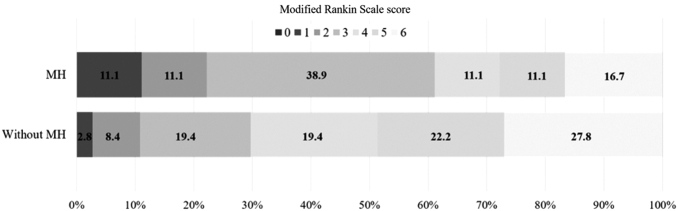
Distribution of mRS scores at discharge in poor-grade SAH patients according to early and prolonged MH. The number indicates the proportion of patients with corresponding mRS score. MH, mild hypothermia; mRS, modified Rankin Scale; SAH, subarachnoid hemorrhage.

According to mRS scores, the number of patients with MH was as follows: mRS score of 1, *n* = 2 (11.1%); mRS score of 2, *n* = 2 (11.1%); mRS score of 3, *n* = 7 (38.9%); mRS score of 4, *n* = 2 (11.1%); mRS score of 5, *n* = 2 (11.1%); and mRS score of 6, *n* = 3 (16.7%) (Fig. 1). Binary logistic regression analysis revealed that early and prolonged MH (OR = 0.156, 95% CI: 0.037–0.644) and severe vasospasm (OR = 5.593, 95% CI: 1.372–22.812) were closely associated with severe functional outcomes at discharge (Table 2).

**Table 2.** Binary Logistic Regression Analysis of Predicting Severe Functional Outcomes in Patients with Poor-Grade Subarachnoid Hemorrhage

Variables	Odds ratio	95% Confidence interval	*p*
Female	1.578	0.329–7.556	0.568
Age	1.046	0.980–1.116	0.175
Hypertension	0.785	0.144–4.272	0.779
Diabetes mellitus	0.836	0.115–6.074	0.860
Hyperlipidemia	2.508	0.384–16.377	0.337
Smoking	2.024	0.159–25.807	0.587
Posterior location	5.911	0.529–66.024	0.149
Aneurysm size (mm)	0.988	0.877–1.113	0.839
Hemoglobin (g/dL)	0.900	0.461–1.760	0.759
SaO_2_ (%)	0.766	0.531–1.106	0.155
Early and prolonged mild hypothermia	0.156	0.037–0.664	0.012
Severe vasospasm	5.593	1.372–22.812	0.016

### Adverse events

We compared adverse events between the two groups within 7 days of admission. We observed more significant shivering in the poor-grade SAH patients treated with MH (*n* = 6, 33.3%) than in those not treated with MH (*n* = 0, 0%; *p* < 0.001). Hypotension tended to be more prevalent in patients with MH (*n* = 4, 22.2%) than in those not treated with MH (*n* = 4, 11.1%), although their difference was not statistically significant (*p* = 0.279). The two groups had no significant difference in the incidence of pneumonia (with MH treatment, *n* = 8, 44.4%; without MH treatment, *n* = 17, 47.2%; *p* = 0.847). However, one patient died in the group receiving MH treatment, despite having received antibiotics. Other variables such as cardiovascular and electrolyte abnormalities did not differ significantly between the two groups (Table 3).

**Table 3.** Comparison of Adverse Events According to Early and Prolonged Mild Hypothermia Within 7 Days of Admission

Variables	Without MH (*n* = 36),* n *(%)	MH (*n* = 18),* n *(%)	*p*
Shivering	0 (0)	6 (33.3)	<0.001
Pneumonia	17 (47.2)	8 (44.4)	0.847
Urinary tract infection	9 (25.0)	5 (27.8)	0.826
Bradycardia	3 (8.3)	2 (11.1)	0.740
Tachycardia	8 (22.2)	6 (33.3)	0.380
Hypotension	4 (11.1)	4 (22.2)	0.279
Seizure	3 (8.3)	1 (5.6)	0.713
Hyperglycemia	9 (25.0)	3 (16.7)	0.487
Hyperkalemia	3 (8.3)	1 (5.6)	0.713
Hyponatremia	12 (33.3)	4 (22.2)	0.399

## Discussion

Our pilot study suggests that early and prolonged MH treatment can help reduce severe functional outcomes in poor-grade SAH patients who undergo coil embolization. Although there were no significant differences in favorable functional outcomes in poor-grade SAH patients as measured by mRS scores of 0 and 2, MH treatment significantly decreased number of patients with mRS scores of 4 and 5. As a result, early and prolonged MH treatment might be associated with a favorable mRS shift to a score of 3, indicating that patients with poor-grade SAH can walk with assistance.

Until now, guidelines as to when to begin treating poor-grade SAH patients with MH have not been set yet. In the past, many studies have focused on the therapeutic efficacy of MH. MH treatment was first tried with intracranial repair of aneurysms. Care should be taken when interpreting results of this study due to heterogeneity across various studies, including differences in SAH severity, neuroprotective drug use, and surgical methods that might affect study results. Todd *et al.* (2005) reported that intraoperative hypothermia (33°C) does not reduce ICU stays, hospitalizations, or mortality as compared with normothermia (36.5°C) in good-grade SAH patients undergoing craniotomy.

Conversely, a meta-analysis including only randomized controlled trials has suggested that intraoperative hypothermia (32–35°C) might reduce mortality and dependency in daily life for good-grade SAH patients, but not in poor-grade SAH patients (Li *et al.*, 2016) Supplemental neuroprotective drugs during temporary clipping have no association with short- or long-term neurological outcomes (Hindman *et al.*, 2010). Based on these results, we can conclude that intraoperative hypothermia does not significantly improve the prognosis of patients with poor-grade SAH.

However, our results showed that early and prolonged MH starting within 8 hours of symptom onset had a therapeutic effect in poor-grade SAH patients undergoing coil embolization. Of course, MH during endovascular coil embolization might have a better effect. However, in reality, it is difficult to do MH while performing coil embolization. More research is needed to confirm therapeutic effects of initiating MH before 8 hours in patients with poor-grade SAH undergoing coil embolization.

How long MH should be continued after SAH is unclear either. Choi *et al.* (2017) have reported that continued use of MH for 48 hours tends to reduce mortality and lead to a good-to-moderate functional outcome, although differences were not statistically significant. Anei *et al.* (2010) have also reported that maintaining MH at 34°C for 48 hours does not improve neurological outcomes in poor-grade SAH patients. These findings suggest that patients with poor-grade SAH might benefit from MH for at least the minimum EBI period.

Multiple pathophysiological mechanisms have been observed during the EBI period, including IICP and decreased CPP, impaired cerebral autoregulation, blood–brain barrier breakdown, cerebral edema, oxidative stress, mitochondrial dysfunction, excitotoxicity, and microglial activation (Rass and Helbok, 2019). ICP in poor-grade SAH patients decreased up to 72 hours. It is then increased again up to 5 days after ictus, as opposed to recovery of brain metabolic distress at 24 hours and brain tissue hypoxia at 48 hours (Helbok *et al.*, 2015). Interleukin-6 is also gradually decreased after 72 hours.

However, it is increased again after 72 hours in patients with DCI (Helbok *et al.*, 2015). Kuramatsu *et al.* (2015) have reported that MH maintaining for an average of 7 days can lead to reduced vasospasm and peak velocity, with a relative risk reduction of 43% in patients with poor-grade SAH. However, severe adverse events associated with prolonged systemic hypothermia occurred in 21% of patients and 6.5% of them died from multiorgan and respiratory failure (Seule *et al.*, 2009). Based on the mentioned results, we selected to maintain MH for 5 days including EBI period and rerising of ICP and inflammatory markers and avoiding adverse events while maintaining MH for 7 days.

Our study revealed that maintaining MH for 5 days did not cause serious adverse events as compared with those without early and prolonged MH. However, shivering was a concern during the induction and rewarming phases of MH, although we used sedative drugs and neuromuscular blockers to manage these complications. Shivering is a normal physiological response to therapeutic hypothermia. Furthermore, shivering itself may reduce the effectiveness of therapeutic hypothermia by increasing the actual metabolic rate and oxygen consumption (Logan *et al.*, 2011).

Thus, shivering should be treated properly to maximize the benefit of therapeutic hypothermia in patients with poor-grade SAH. Kimberger *et al.* (2007) have reported that combining meperidine and skin warming can effectively decrease shivering thresholds. Future research should investigate whether early and prolonged MH can effectively treat shivering in patients with poor-grade SAH using pharmacological and nonpharmacological protocols.

This study has some limitations. First, all enrolled patients had undergone coil embolization. In clinical practice, it is difficult to perform MH within 8 hours after SAH onset because of the time required for surgical clipping. Accordingly, results of this study might not be reproducible in poor-grade SAH patients who have undergone surgical clipping. Second, we implemented MH using surface cooling blankets connected to a Medi-Therm III Hyper/Hypothermia Machine MTA6900.

Although there were no significant differences in reaching a target temperature among standard blankets, ice packs, and the Arctic Sun^®^ (Medivance Corp., Louisville, CO, USA), the Arctic Sun cooled patients more rapidly than standard cooling blankets (Heard *et al.*, 2010). Arctic Sun is known to have higher cold fluid flow rates and a precise temperature feedback-control system (Heard *et al.*, 2010). Thus, it is likely to be more effective in managing early and prolonged MH in poor-grade SAH patients. Third, SAH patients from two institutions participated in this study. Neurological outcomes of SAH patients differed between institutions as well as countries.

In addition, patients' characteristics and timing of SAH treatment were somewhat different among institutions (Dijkland *et al.*, 2019). Therefore, it is necessary to conduct a randomized controlled trial that targets a large number of patients in the future. In particular, in the case of multicenter studies, strict control of factors (e.g., sedation, blood pressure, and IICP) that can affect treatment outcomes through a common protocol is required (Alotaibi *et al.*, 2017; Darkwah Oppong *et al.*, 2022). It is necessary to analyze adverse events of MH including the rewarming phase and the hypothermia period (Supplementary Tables S1 and S2).

## Conclusion

This pilot study confirmed the possibility of therapeutic effects of early and prolonged MH in patients with poor-grade SAH. A randomized controlled study with a large number of patients is required to confirm our findings in the future.

## Supplementary Material

Supplemental data

Supplemental data

Supplemental data
