# Supplementary material for: Early and Prolonged Mild Hypothermia in Patients with Poor-Grade Subarachnoid Hemorrhage: A Pilot Study
Source: Ther Hypothermia Temp Manag. 2022 Nov 25;12(4):229–34. doi: 10.1089/ther.2022.0013 (PMC9700366; doi:10.1089/ther.2022.0013)

**Supplemental Figure S1.** Chronological changes in mean body temperature between mild hypothermia (MH) and non-MH groups. Data are presented as mean with 95 % confidence interval.


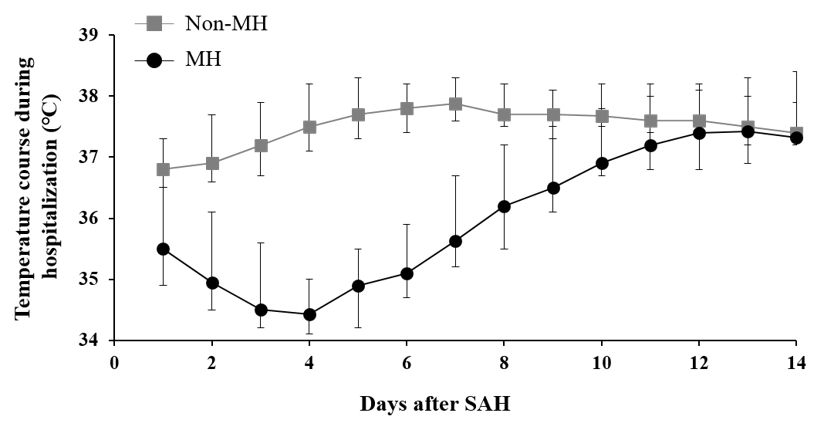

Supplement: Supplemental data [file Suppl_FigS1.docx]
