# Supplementary material for: Early and Prolonged Mild Hypothermia in Patients with Poor-Grade Subarachnoid Hemorrhage: A Pilot Study
Source: Ther Hypothermia Temp Manag. 2022 Nov 25;12(4):229–34. doi: 10.1089/ther.2022.0013 (PMC9700366; doi:10.1089/ther.2022.0013)
Supplement: Supplemental data [file Suppl_TableS1.docx]

**Supplemental Table S1.** Further analysis of differences in factors that might affect patient outcome according to mild hypothermia (MH).

| **Variables** | **Without MH (n = 36)** | **MH (n = 18)** | ***p*-value** |
| --- | --- | --- | --- |
| Sedation | 17 (47.2%) | 11 (61.1%) | 0.336 |
| Increased mean arterial pressure | 21 (58.3%) | 11 (61.1%) | 0.845 |
| Increased intracranial pressure | 23 (63.9%) | 13 (72.2%) | 0.540 |
| electrolyte imbalance | 15 (41.7%) | 8 (44.4%) | 0.846 |
