# Supplementary material for: Early and Prolonged Mild Hypothermia in Patients with Poor-Grade Subarachnoid Hemorrhage: A Pilot Study
Source: Ther Hypothermia Temp Manag. 2022 Nov 25;12(4):229–34. doi: 10.1089/ther.2022.0013 (PMC9700366; doi:10.1089/ther.2022.0013)
Supplement: Supplemental data [file Suppl_TableS2.docx]

**Supplemental Table S2.** Comparison of adverse events according to early and prolonged mild hypothermia (MH) within 14 days after subarachnoid hemorrhage.

| Variables | Without MH (n = 36) | MH (n = 18) | *p*-value |
| --- | --- | --- | --- |
| Shivering | 0 (0%) | 7 (38.9%) | <0.001 |
| Pneumonia | 19 (52.8%) | 11 (61.1%) | 0.561 |
| Urinary tract infection | 11 (30.6%) | 8 (44.4%) | 0.314 |
| Bradycardia | 4 (11.1%) | 3 (16.7%) | 0.567 |
| Tachycardia | 10 (27.8%) | 8 (44.4%) | 0.221 |
| Hypotension | 5 (13.9%) | 5 (27.8%) | 0.215 |
| Seizure | 3 (8.3%) | 1 (5.6%) | 0.713 |
| Hyperglycemia | 11 (30.6%) | 5 (27.8%) | 0.833 |
| Hyperkalemia | 4 (11.1%) | 3 (16.7%) | 0.567 |
| Hyponatremia | 14 (38.9%) | 8 (44.4%) | 0.695 |
